# User evaluation of a therapist-guided internet-delivered treatment program for anxiety disorders: A qualitative study

**DOI:** 10.1016/j.invent.2021.100389

**Published:** 2021-04-18

**Authors:** Hege Mari Johnsen, Kristine Haddeland

**Affiliations:** Department of Health and Nursing Science, Faculty of Health and Sport Sciences, University of Agder, Norway

**Keywords:** Anxiety disorders, Cognitive behaviour therapy, Evaluation, ICBT, Information systems success model, Primary health services

## Abstract

Therapist-guided internet-based cognitive behaviour therapy (ICBT) has been proposed as a potential means to increase individuals' access to quality mental health care and effective treatment. Guided ICBT aims to increase a patient's knowledge and competence to better cope with their disorder. Despite the growing evidence supporting the effects of guided ICBT, there is remarkably little research on the different factors that are important for patients to achieve effects from using such digital treatment interventions. Thus, the aim of this study was to conduct a user evaluation of a therapist-guided ICBT program using the updated DeLone and McLean (D&M) model for measuring information systems (IS) success or effectiveness. This model includes the following six dimensions: system quality, information quality, service quality, intention to use and use, user satisfaction, and net benefits (impacts or effect).

Ten users of a Norwegian therapist-guided ICBT program for treating anxiety disorders named ‘*Assisted Self*-*Help*’ (Assistert Selvhjelp) participated in phone-based individual interviews. Data were analysed using directed content analysis. Results showed that the participants were quite satisfied with the program's system quality and information quality. However, participants suggested improvements, including in-program instruction, improved visibility of system status, more flexibility regarding automated measurement surveys, and the inclusion of more videos with patient stories. Further, the results indicated a need for improvement in the service quality of guided ICBT introduction, instruction, follow-up, guidance, and support from therapists. The results showed that user friendliness and high educational content might not be sufficient for a therapist-guided ICBT program to be perceived as effective. It might also be necessary for therapists to provide follow-up, guidance, and support that are more in line with individual patient needs. Thus, the results suggest that guided ICBT requires active participation from all involved in the process, including the therapist.

## Highlights

• The Norwegian Assistert Selvhjelp ICBT for treating anxiety disorders was assessed.
• User evaluations were conducted with 10 patients based on the DeLone and McLean Model of IS Success.
• Overall, patients were satisfied with the internet-based cognitive behaviour therapy program.
• Improvements in follow-up, guidance, and support from therapists were requested.
• Guidelines should ensure the implementation of various important success dimensions.

## 1 Introduction

Anxiety disorders are common mental health disorders and are a major public health concern worldwide (National Institute of Mental Health, 2020). There are many types of anxiety disorders, including panic disorder, agoraphobia, social phobia, posttraumatic stress disorder, acute stress disorder, generalized anxiety disorder, obsessive compulsive disorder, and specific phobia. These disorders can include *physical* symptoms, such as trembling, tense muscles, or rapid breathing; *cognitive* symptoms such as worries and difficulty concentrating; *emotional* symptoms, such as distress or irritability; and *behavioural* symptoms, such as difficulty sleeping (Olthuis et al., 2016). Social phobia and panic disorder constitute two of the most common psychiatric disorders in the general population (Pedersen et al., 2020). These disorders may lead to impaired functioning in daily life and are generally treated with cognitive behavioural therapy (CBT), medication, or both (National Institute of Mental Health, 2020).

Cognitive behavioural therapy (CBT) is an evidence-based treatment for anxiety disorders (Olthuis et al., 2016). CBT is based on the cognitive model of mental illness initially developed by Beck (1964). This cognitive model hypothesises that people's emotions and behaviours are influenced by their perceptions of events. For example, depressed patients are considered excessively negative in their interpretations of events. As its name suggests, CBT includes both cognitive and behavioural interventions or techniques. Unfortunately, certain barriers (for example, time constraints, a lack of sufficiently qualified clinicians, stigma or long waiting lists) continue to limit access to CBT (Olthuis et al., 2016; Mohr et al., 2006). Therapist-guided internet-based cognitive behaviour therapy (ICBT) is an approach wherein CBT is delivered over the internet, with a therapist providing guidance asynchronously (via email or text chat) or synchronously (via phone call, face-to-face consult, or video consult). The structure of guided ICBT resembles regular face-to-face treatment in content evidence-based knowledge about anxiety, symptom assessment, and assignments (i.e., thought diaries and exposure techniques). Such programs contain audio, videos, and animations. Patients may read the content at their own pace and practice and apply exposure techniques in their daily lives. Use of therapist-guided ICBT is one way to minimize treatment barriers, thereby increasing access to treatment (Karyotaki et al., 2019).

The goal of the World Health Organisation's Special Initiative for Mental Health (2019–2023) is to ensure universal health coverage of mental health conditions (World Health Organisation, 2019). One of the initiative's strategies involves scaling up interventions and services across community-based, general health, and specialist settings. In addition to empowering communities to provide the highest standard of health care, it is important to empower individuals to ensure their own mental health and well-being (World Health Organisation, 2019). This initiative aligns with the third United Nations Sustainable Development Goal: to ensure healthy lives and promote well-being for all people, regardless of age (United Nations, 2020).

The effect of therapist-guided ICBT has been investigated in many randomized controlled trials (Ciuca et al., 2018; Jones et al., 2016; Andersson et al., 2012), as attested by systematic reviews and meta-analyses (Polak et al., 2021; Andrews et al., 2018; Carlbring et al., 2018; Olthuis et al., 2016). Despite the growing interest in studies measuring the effect of guided ICBT, few studies have explored patient's experiences with using it. In a meta-synthesis of common themes in user experiences across eight qualitative studies involving ICBT programs for depression and anxiety disorders delivered with minimal or no professional support, the researchers reported two main findings (Knowles et al., 2014). The results identified the need for treatments to be sensitive to the individual user and both negative and positive consequences of adding additional support to the treatment (Knowles et al., 2014). Thus, while adding more support seemed to make ICBT more acceptable to some users who found it difficult or less meaningful to work with online materials on their own, others preferred being able to work more independently with their problems. In a recently published qualitative study of patients' experience of ICBT programs with minimal support (Pedersen et al., 2020), a large majority of the participants expressed a need for additional support and face-to-face contact with a clinician. In another qualitative study by Walsh and Richards (2017), evaluating user's experiences and engagement with the design features of a guided ICBT program for anxiety, participants received a weekly review of their progress from an online supporter. The results of this study elaborated on the importance of guidance alongside as a key to the success of internet-delivered interventions (Walsh and Richards, 2017). The importance of therapeutic alliances to increase treatment outcomes has also been identified in other studies (Pihlaja et al., 2018; Shim et al., 2017; Stjerneklar et al., 2019).

Service delivery models for guided ICBT programs and technologies differ, and there are examples of studies in which effects have been minimal or where findings contradict expectations (Kaldo et al., 2013). This may be explained by the fact that few guided ICBT programs have been tested for usability (Yogarajah et al., 2020) or have been put through an evaluation that includes individual, technical, and organisational factors (Health Quality Ontario, 2019). The effects or impacts of information technology (IT) are often indirect and may be influenced by technological, human, organisational, and environmental factors. This should be considered when measuring the success of information systems (Petter et al., 2008). The updated DeLone and McLean (D&M) model for measuring IS success or effectiveness assesses IS success by evaluating six dimensions: system quality, information quality, service quality, intention to use and use, user satisfaction, and net benefits (Delone and McLean, 2003). It is a widely used framework for predicting and evaluating the success of IS. A critical meta-review conducted by Jeyaraj (2020) shows that the D&M framework has been empirically tested and employed in measuring the IS success of many information systems, internet-based systems such as e-government systems, online shopping, online communities, e-commerce, and social networking. However, similar to Jeyaraj (2020), we did not find any studies that used the D&M framework to evaluate the success of guided ICBT in our literature search conducted in Medline and CINAHL. Consequently, the aim of this study was to conduct a user evaluation of a therapist-guided ICBT program (‘*Assisted Self*-*Help*’) for treating anxiety disorders using the updated D&M model for measuring IS success or effectiveness (Delone and McLean, 2003).

## 2 Materials and methods

### 2.1 Research model

**Fig. 1:**
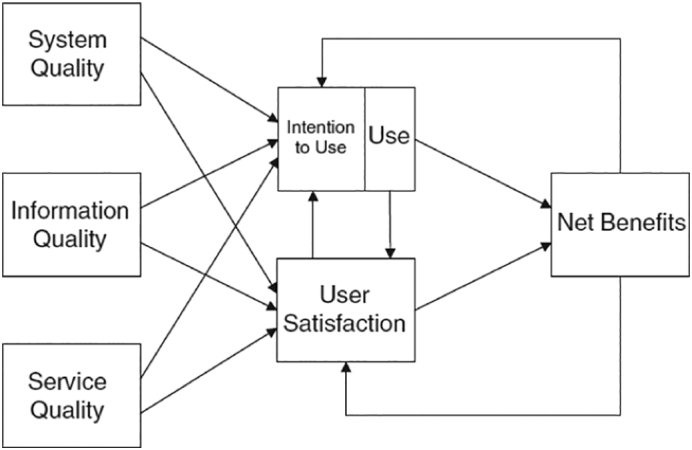
The updated D&M IS success model (Petter et al., 2008, p. 238).

This study is guided by the updated D&M model for measuring IS success or effectiveness (Delone and McLean, 2003). This model considers IS success to be a multidimensional and interdependent construct. As shown in Fig. 1, the updated D&M model includes six dimensions: system quality, information quality, service quality, intention to use and use, user satisfaction, and net benefits.

To demonstrate the proposed associations among the dimensions, the model includes several arrows. The quality dimensions (information quality, system quality, and service quality) will singularly or jointly affect subsequent “use” and “user satisfaction”. Intention to use, use, user satisfaction, and net benefits measure effectiveness success. For example, attitudes (intention to use) links with behaviour (use). Positive experiences with “use” will lead to greater “user satisfaction” and further to increased “intention to use”. Certain “net benefits” will occur as a result of “use” and “user satisfaction”. Experience of positive or negative benefits will lead to increased or decreased intention to use and user satisfaction (Delone and McLean, 2003). The dimensions and examples of measures for IS success suggested by Delone and Mclean (2003) and Petter et al. (2008) are shown in Table 1.

**Table 1:** The six dimensions of the DeLone and McLean (D&M) model of information systems (IS) success and examples of measures for IS success.

Dimensions of the D&M IS success model	Examples of measures for IS success
1. System quality	Ease of learning, ease of use (usability), intuitiveness, system flexibility, availability, system reliability and desirable characteristics.
2. Information quality	Ease of understanding, relevance, completeness, accuracy, conciseness, personalization and security.
3. Service quality	Support delivered by the service provider. This could entail IT support but also other types of user support and empathy from personnel staff.
4. Intention to use and use	Attitude towards using and re-using the system. Appropriateness of use, frequency of use, time of use, usage patterns, dependencies, and purpose of use.
5. Satisfaction	Overall opinions about the system.
6. Net benefits	Impacts on individual users, groups, organisations, industries, and nations.

According to Delone and McLean (2003), the application context of the model should dictate the appropriate specification and application.

### 2.2 The therapist-guided ICBT program for treating anxiety

The therapist-guided ICBT program ‘*Assisted Self*-*Help*’ (Assistert Selvhjelp) for treating anxiety, is a low-threshold mental health service for treating mild-to-moderate anxiety disorders among people over 16 years of age. ‘*Assisted Self*-*Help*’ is distributed as a public healthcare service, mainly by the mental health services in municipalities, which focus on short-term early interventions. People may be referred through their general practitioner (GP), rapid municipal mental health services, or student organisations. Therapists who offer this program have backgrounds mainly in health or social work and are typically nurses, psychologists, or social workers. Most of these therapists have additional training in cognitive behaviour therapy.

**Fig. 2:**
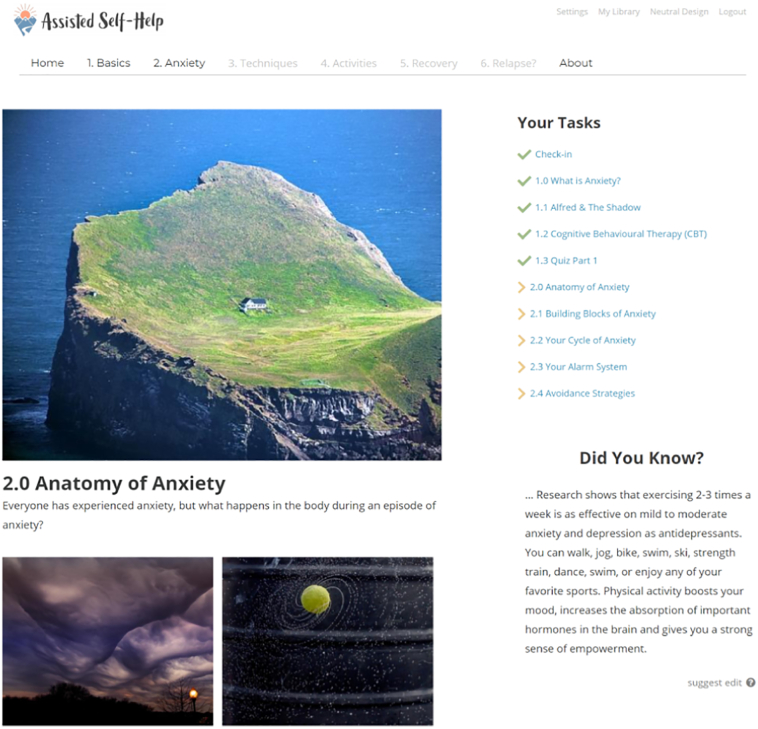
A screenshot of the user interface of the program for anxiety disorders.

**Fig. 3:**
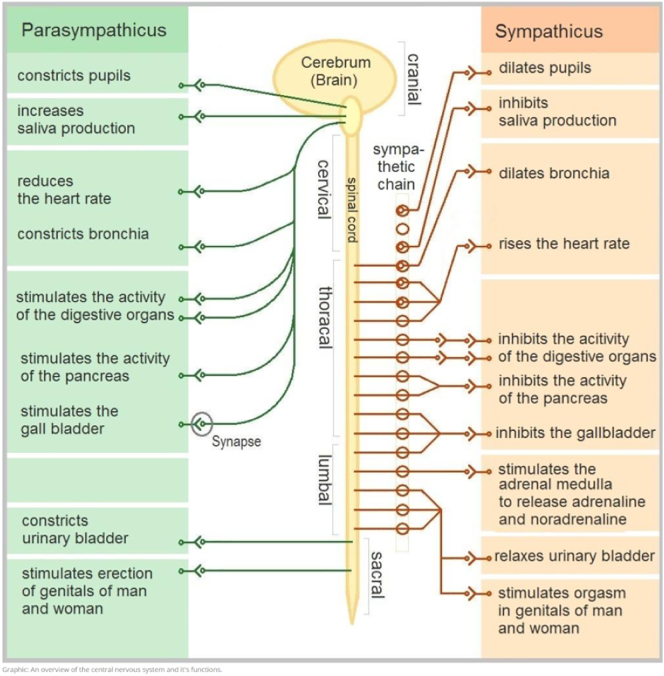
A screenshot of the physiological explanation of anxiety disorders.

The ‘*Assisted Self*-*Help*’ program involves exercises, tasks, and information based on how a psychologist would design an anxiety therapy process. This program follows a fixed structure with the following six modules: 1) “What is Anxiety?”, 2) “The Physiology of Anxiety,” 3) “Basic Techniques,” 4) “What Kind of Anxiety Do You Suffer From?”, 5) “Practice Makes Perfect,” and 6) “Setback or Relapse?”. After the first three modules that generally apply to all anxiety disorders, patients enter the fourth module, in which one of the four following types of anxiety is chosen based on the patient's primary issue: social situations (social anxiety), anxiety attacks (panic anxiety), specific anxiety (phobic anxiety), or anxiety in various situations without a particular cause (general anxiety). The patients received guidance from the therapist on this part. The rest of the program is adapted to the user's type of anxiety. No personal data that can directly identify the user are stored. The program is provided on personal computers, tablets, and smartphones. The whole program could be finished within 6 weeks. Figs. 2 and 3 show screenshots from the program for anxiety disorders.

The participants in our study had mostly been referred by their GP or through rapid municipal mental health services. After the therapist deemed a user suitable for this form of treatment (i.e., having a mild-to-moderate anxiety disorder and the ability to work independently), the user received access to the program with a unique and anonymized code. Regular structured follow-ups were conducted by therapists via individual in-person or online meetings (lasting up to 45 min), and/or by telephone consult (lasting 10–20 min). Patients were offered weekly consultations.

Little empirical research has been conducted involving the ‘Assisted Self-Help’ therapist-guided ICBT program. The program has been included in a study that evaluated the usability of five Scandinavian therapist-guided ICBT programs conducted by Yogarajah et al. (2020). According to the developers of ‘Assisted Self-Help,’ a randomized controlled trial (RCT) is under planning in collaboration with the Norwegian Institute of Public Health.

### 2.3 Study design and interview method

This study used an explorative qualitative design. User evaluations of the ‘*Assisted Self*-*Help*’ program for treating anxiety disorders were conducted. Here, evaluation is defined as “the act of measuring and exploring properties of a health information system, the result of which informs a decision to be made concerning that system in a specific context” (Ammenwerth et al., 2004, p. 408). A purposeful sample of ‘*Assisted Self*-*Help*’ users participated in individual phone-based semi-structured interviews. The interview guide contained 20 questions. The interview started by gathering some patient demographics, such as age, gender, and previous experience with similar programs. Further, in line with Hsieh and Shannon's (2005) directions for a deductive approach, the interview guide continued with open-ended and targeted questions guided by the six dimensions of the updated D&M model presented in Table 1. However, as suggested by Delone and McLean (2003), the questions were dictated by the ICBT context. Table 2 shows how the authors have applied the D&M model in the development of questions for the user evaluation of the guided ICBT program ‘*Assisted Self*-*Help*’.

**Table 2:** Examples of interview questions guided by the updated D&M IS model.

Dimensions of the D&M IS model	Examples of interview questions
1. System quality	How did you experience the user-friendliness of the ‘*Assisted Self*-*Help*’ program? Desired functionality? How did you experience the characteristics of the user interface design?
2. Information quality	Was the information content easy to understand? (why/why not) What about the relevance of its content?
3. Service quality	How was ‘*Assisted Self*-*Help*’ introduced to you? Did you receive instructions and guidance on how to use the program? (before use/when using) How was follow-up and support during use?
4. Intention to use and use	Did anything in particular affect your motivation and intention to use the program or its perceived usefulness? How frequent and how much time have you used on your own efforts (more or less)? How does the use of the program correspond with the agreement made with your therapist? Do you have any preferences for how to use the program?
5. Satisfaction	What are your opinions about the ‘*Assisted Self*-*Help*’ program?
6. Individual impact	What has been the user value of this program to you? Has the solution contributed to increased insight into and coping with your anxiety disorder? (why/why not?)

The interview guide was tested by two specialists in mental healthcare who has also been involved in developing the program. They were asked to comment on relevance of questions and encouraged to suggest additional questions. This resulted in two additional questions, one about the actual use of the program and one about the follow-up by therapists. The two specialists approved the final version of the interview guide before the data collection started. However, they did not take part in any other parts of the study.

The interviews were conducted on the phone by the main author and lasted 18–50 min, with an average duration of 30 min. The interviews were recorded with a separate offline audio recorder.

### 2.4 Participants and recruitment

A purposeful sampling method was used. Patients were recruited from three different municipalities and six different therapists. The unit manager in each municipality contacted their respective therapists, who further recruited participants based on the following criteria: 1) mild-to-moderate anxiety disorder, 2) normal cognitive function, and 3) ability to work independently. It was preferable to the study design that participants be over 18 years old, there be a spectrum of ages and genders, and participants have completed the whole or at least half of the ‘*Assisted Self*-*Help*’ program. No data were collected regarding how many patients were invited to participate and declined.

According to Polit and Beck (2010), the number of participants needed for a qualitative study depends on the aim of the study. For an evaluation of information systems, eight to ten participants may provide sufficient data if the participants selected are representative of the target users of the system being assessed (Kushniruk and Patel, 2004). As a result, ten patients who had used the ‘*Assisted Self*-*Help*’ program for treating anxiety disorders were recruited for this study, and they were contacted by the main author to schedule time for the interview via phone. The main author and the participants had not been in contact before this.

### 2.5 Data analysis

A professional service transcribed all the interviews. The main author who conducted the interviews read through the transcribed data and compared them with the audio data. According to Hsieh and Shannon (2005), existing theory can help focus the research question and determine the coding scheme by using a directed content analysis. Because this study aimed to conduct a user evaluation of the ‘*Assisted Self*-*Help*’ program guided by a theoretical framework for measuring IS success or effectiveness (Table 1), the analysis process was inspired by Hsieh and Shannon's (2005) suggestions for a directed content analysis. The analysis followed these three steps:

1) First, both authors read all textual data and highlighted parts of the text that, on first impression, appeared to be related to the six dimensions dictated by the D&M framework. These dimensions were viewed as predetermined main categories for the analysis.
2) Second, the first author used NVIVO 12 software (QSR International, 2020) for data management and initial coding. All the transcribed interviews were initially uploaded into NVIVO. Then, a coding matrix of the six main categories was created in NVIVO, followed by the coding of the textual data into the six categories. Next, the text sorted under each category was reread inductively to identify possible subcategories that could fit any of the suggested examples of measures suggested in Table 1.
3) Third, the two authors met to discuss the fit between the textual data and its highlighted parts with the coding of main- and sub-categories. The two authors did not identify any highlighted text that had not been coded into the six main categories. However, some of the identified subcategories did not fit the suggested measures by D&M (Table 1) and were adjusted to the ICBT context by the authors.

### 2.6 Ethical considerations

This study was approved by the Norwegian Center for Research Data (Number: 214266) and followed general ethical guidelines (The Norwegian National Research Ethics Committees, 2014). Since the aim of this study was to conduct a user evaluation of the therapist-guided ICBT program ‘*Assisted Self*-*Help*’, and not to measure the effects on the user's anxiety symptoms, more detailed data regarding the patient's mental health condition were not collected. Therefore, approval from the Regional Committees for Medical and Health Research Ethics was not required (Number: 117187).

All participants received written and oral information about the project, and oral consent was acquired and recorded before the interview started. Each participant's name was assigned a unique number in the event of requirement of further information. Confidentiality was maintained by storing names and participant numbers separately and by storing all personal information in a secure location.

## 3 Results

The characteristics of the 10 participants are displayed in Table 3. Completion time of the ‘*Assisted Self*-*Help*’ program varied from one to six months, with two months being the most common completion time.

**Table 3:** Participant demographics (*n* = 10).

Gender N (%)	
Female	6 (60)
Male	4 (40)
Age years	
Mean, range	31 (21 to 53)
Previous experience with similar ICBT programs (%)	
Yes	3 (30)
No	7 (70)
Completion of the ‘Assisted Self-Help’ program (%)	
Completed the program	5 (50)
Completed more than half	4 (40)
Completed less than half	1 (10)

The participants that had prior experience with similar online resources had used other programs within the broader ‘*Assisted Self*-*Help*’ collection of resources, such as for stress, worry, and depression. Two participants used other types of online resources, such as apps and YouTube videos. Table 4 provides the main findings identified in this study, including an example of one quote from each main category. Description of the results related to each subcategory will be further described in the text.

**Table 4:** An overview of the main findings of this study.

Main categories	Subcategories	Examples of quotes
1. System quality	Ease of use System flexibility Desirable characteristics	“…it was quite self-explanatory”
2. Information quality	Ease of understanding Relevance of content	“When you panic, there is something about having such tools in the back of your head”
3. Service quality	Introduction to the program Information support Follow-up and guidance	“She just asked me if I would try it, and ultimately just sent me a code”
4. Intention to use and use	Attitude towards using and re-using the program Patterns of usage	“I have not spent much time with the program itself, but have spent a lot of time reflecting on the content”
5. User satisfaction	Opinions Perceived advantages and disadvantages	“I think it is a very nice and straightforward program that contains a lot of useful information. So, I am very impressed”
6. Individual impacts	Gain of knowledge Competence to cope	“I feel things have gone much better in the last few weeks after I started with it, absolutely”

### 3.1 System quality

#### 3.1.1 Ease of use

Only one participant thought it was cumbersome to log in due to difficulty remembering the code. That participant expressed, “It should have been possible to create a personal code when logging in for the first time” (Participant 5). Further, nine out of ten participants thought learning to use the program was easy. One expressed, “…it was quite self-explanatory” (Participant 10). However, two participants proposed that the program should have included information or an instructional video on how to use it. One participant missed information about whether to complete the modules in order or whether it was possible to complete the modules, depending on personal needs. Only two participants had experienced technical issues. One had been disconnected from the program, but was unsure whether that was due to the program itself or the participant's internet access. The other participant experienced problems accessing a YouTube video in one of the modules.

Due to information security issues, the participants could not fill out the forms within the program. The forms either had to be downloaded or printed. As a result, one of the participants chose not to use these forms. However, the other participants downloaded and printed them out or just used the forms to take notes elsewhere. One commented that the forms were greatly beneficial because they provided an overview of the mental health situation.

A participant's progress in the program is indicated by colour codes for complete and incomplete modules. One participant (who used an iPhone) thought it was hard to navigate the overview of the progress made in the program. Another participant stated that if an entire module was not completed in one sitting, one had to start over again the next time instead of being able to resume the progress of the prior attempt. Further, one participant expressed that it was difficult to know approximately how long each module took to finish and how many tasks remained before finishing a module. The user interface displayed participant progress in percentage but did not show how many tasks were left.

#### 3.1.2 System flexibility

Two participants expressed that they did not like the protocol of being forced to fill in a questionnaire containing four to five questions regarding their psychological condition and social relations before each module. One participant experiencing stress stated, “I do not always feel like answering these questions” (Participant 6). The same participant suggested that the questions be asked at the end of each module. The other participant thought being forced to answer these questions before each module was inconvenient, especially after being asked similar questions in several modules in a row. In contrast, another participant commented that it was nice to be able to follow one's own progress by looking at a diagram.

One participant thought the program became complicated to use when there were forms that had to be filled out about situations where the participants felt insecure: “It's probably what you have to do to get rid of your problems, but it did not quite suit me” (Participant 5).

Most participants appreciated that they could go back and forth in the program by using arrows. However, one participant expressed that the flexibility of being able to automatically move to the next module could be a drawback, as she read too much too fast. Another participant suggested including a function that asked if you wanted to move on to the next module.

#### 3.1.3 Desirable characteristics

Most participants expressed that they enjoyed the user interface design, which employed a combination of images, videos, animations, and quizzes. Some of the comments included: “nice pictures”, “text is tidy and has proper size”, “simple and minimalist”, and “clever with video clips in between”. One participant elaborated especially on the importance of videos in the program: “I thought the opportunity to watch videos between text and questions was great” (Participant 1). However, two participants commented that they thought the animations used were too juvenile. Similarly, one participant commented that the animations were not particularly educational. However, almost all participants expressed that they appreciated the videos. Only one participant reported, “I think it is better to read text than watching the videos, because I easily lose concentration when I watch videos” (Participant 8). By contrast, another participant reported greater benefits from videos and audio files than from large amounts of text. Two participants suggested including more videos in which people with anxiety disorders share their experiences, as this may help participants self-identify.

### 3.2 Information quality

#### 3.2.1 Ease of understanding

Examples of comments provided about the program's information quality include: “The information was well explained”, “appropriate amount of information”, and “easy to read and understand”. However, one participant of foreign origin struggled to understand some of the content, especially the exposure techniques and exercises. The participant in question received explanations from the therapist when needed.

Regarding the structure of the information, most participants agreed that it was logical. One participant commented that it was beneficial that there were different modules containing unique tasks that helped participants focus on one thing at a time. Two participants also commented that they appreciated how the program started simply and became more advanced over time. It was also noted that it was helpful that each module ended with a quiz to test whether the participant understood the information. If participants realised that they had not understood, they could return to the reading for clarity.

#### 3.2.2 Relevance of content

Participants commented that the program had an “appropriate amount of information”. Regarding the relevance and usefulness of the program's content, one participant felt that the program had not been very informative, as the participant already had substantial knowledge. However, the participant in question acknowledged that the program provided useful information for those who did not have as much knowledge of the field. Another participant who had prior knowledge thought it was helpful to have this knowledge refreshed. One other participant commented that it was beneficial that the program provided repetition of certain content, especially for users who had minimal prior knowledge.

As mentioned in Section 3.1.3, it was appreciated that the program contained real-life stories told by people with anxiety disorders. As one participant expressed, “I have missed that, because I kind of felt like the only one in the whole world who has it like this” (Participant 3). Several participants commented that they appreciated the theoretical explanations related to different anxiety disorders. One participant commented: “I think it is very nice when there are explanations of what happens in your body and what causes the symptoms, and explanations of the different symptoms” (Participant 2). Further, one participant pointed out that it was reassuring and important that the content in the program was created by professionals. One participant commented that the section entitled “Did You Know That?” was especially useful because it included facts about anxiety disorders. It was also appreciated that the program provided guidance and tips on how to deal with personal anxiety disorder. One participant said, “When you panic, there is something about having such tools in the back of your head” (Participant 3).

Some participants commented that it was difficult to choose only one of the anxiety categories because they felt that either a single category did not represent everything they recognized in themselves or that multiple categories were applicable. Some commented that they used other online resources and apps in addition. Participants also noted that it was helpful to have access to other programs in the broader ‘*Assisted Self*-*Help*’ resource (e.g., stress and depression).

### 3.3 Service quality

#### 3.3.1 Introduction to the program

The ‘*Assisted Self*-*Help*’ was recommended to three of the participants by a GP, other healthcare workers or friends. As one participant was unsuccessful in searching for information about the program on their municipality's website, this participant recommended that information about such a resource be made available and more open to the public. It was suggested that such access could decrease the stigma associated with mental health disorders and make it easier for people to seek help with their disorder.

The ‘*Assisted Self*-*Help*’ guided ICBT program was mostly introduced to participants by the therapists as a resource they could use at home to apply the skills and strategies learnt in the program to better cope with their own personal situation. One participant expressed, “I didn't quite understand what the therapist was talking about, but I got the urge to try it” (Participant 2). One participant also commented that the therapist had been very limited with information in the introduction of the program: “She just asked me if I would try it, and ultimately just sent me a code” (Participant 4). Another participant was only offered the opportunity to use the program because they had tried all other available treatment options in the municipality.

One participant was introduced to the ‘*Assisted Self*-*Help*’ resource as an alternative to regular treatment while the participant was on a waiting list for regular treatment. The participants were offered the use of the program until a regular appointment with the therapist was available.

#### 3.3.2 Information support

Nine participants commented that they had to find out for themselves how the program worked and were sent only a code for entering the program. Only one out of the 10 participants had received a demonstration of the program from their therapist. One participant expressed, “I feel it would have been much easier if I my therapist and I had done it together the first time, that I had been shown a little how it works” (Participant 3). Another participant lamented not receiving information about the full content of the program. For example, this participant was unaware that exposure tasks were included in the program. The participant said, “If the therapist had given me more information about the program and had been more motivating, I would have benefited more from the program” (Participant 4). The kind of instruction the participants received from their therapists regarding how to use the program or how much material to complete within a given period varied greatly. Only half of the participants thought they had been given clear instructions on how much to complete between appointments. One participant proposed that the therapist should be allowed to access the full program to be able to check user progress. Two participants thought that the lack of information and instructions might have been due to the therapist not receiving any special training in using the program or not having spent the time becoming familiar with the content.

#### 3.3.3 Follow-up and guidance

Four participants had appointments with the therapist once a week, two had appointments every other week, and three had irregular appointments. One participant did not have appointments in the beginning but eventually secured appointments after being assigned a therapist. It was then agreed that the participants would meet with the therapist once a week. No participants expressed that they needed more frequent meetings than what was agreed upon. Two of the participants expressed that the most important was how time was spent during the appointments with your therapist.

Six of the participants had only telephone contact with their therapists. Two initially had telephone contact but were offered digital video meetings after expressing the need for in-person meetings with their therapist. Physical meetings could not be conducted due to the COVID-19 pandemic. As a result, two of the participants who initially had physical meetings with their therapists had to switch to telephone contact. One expressed, “I feel it would have been much better if I could have come to the therapist's office once a week instead of just calling” (Participant 5).

Three participants were not satisfied with the guidance or support they received from their therapists while using the program. One of the participants commented that while there was nothing wrong with the program itself, the follow-up, guidance, and support were insufficient. This participant recommended more assistance with carrying out the exposure therapy, as this was perceived to be the most important segment of the program: “You have been given tools, but do not always know how to use them” (Participant 3).

Further, one of the participants was unclear on what extent of the program was the therapist's responsibility versus the participant's. All three participants mentioned issues including a lack of specific assignments from one appointment to the other, minimal or no follow-up conversation after each module, and failure to set up an exposure plan together with the therapist.

Among the participants who were satisfied with the support and guidance from their therapist, three expressed that they found the briefing with their therapist particularly useful, as they could review and discuss what they had completed in the program and receive explanations on a segment that was difficult to understand. One of the participants reported improved understanding through the combination of using the program and having these subsequent conversations with the therapist. One participant did not require much follow-up due to significant progress alone. That participant did, however, remark that it was reassuring to be able to schedule an occasional check-in with the therapist and be reminded to work on the program further. Some participants commented that it was useful to be assigned specific tasks and to be actively encouraged by their therapist. One remarked: “She got strict with me and said I had to use ten minutes each day to work on the program, then I just had to grab myself by the neck and do it” (Participant 10).

### 3.4 Intention to use and use

#### 3.4.1 Attitudes towards using and reusing the program

Three of the participants expressed that they were not particularly motivated by the introduction provided by their therapists. One of the reasons mentioned for the lack of motivation was that the therapist did not have enough knowledge about the program. Those who felt that follow-up from the therapist had been sufficient remarked that the therapist had managed to motivate them and positively impact their success. All 10 participants expressed that the follow-up and guidance (or lack thereof) provided by their therapists affected their efforts as well as their perceptions of the program's usefulness.

Nine out of 10 participants stated that they would continue to use the program even after they had completed it. One participant expressed a wish to complete the entire program one more time, while another only wanted to review certain sections and use the techniques learned. Participants viewed access to the program for a whole year and access to the other programs in the online resource as positive. Several participants expressed that they wanted to use the other programs within the ‘*Assisted Self*-*Help*’ resource.

#### 3.4.2 Patterns of use

The participants' use of the program varied in both the number of hours spent per week and the minutes spent in each sitting. Six of the participants could not estimate how many times per week they used the program, as it varied greatly. Four of the participants stated that they used the program approximately one or two times per week. The estimated time spent using the program in a single sitting varied from 10 min to 3 h. One of the participants commented, “I have actually used it whenever I have felt for it myself” (Participant 1).

Six of the participants used the program in accordance with an agreement with their therapists. Two participants had used it over a longer period of time than agreed upon due to a lack of time or opportunity to sit down undisturbed. Among the two remaining participants, a lack of follow-up and reduced motivation were given as reasons for their level of use; these participants were among those who were not satisfied with their follow-ups.

Participants were asked if they had any preferences for how the program should be used to achieve the best results from this kind of treatment. One participant stated that one had to be undisturbed when using the program, as it was quite energy intensive. Another participant commented that the most important thing was getting started with the program. It was recommended as part of a regular routine but not excessively during one sitting (i.e., no more than 15–30 min). One participant suggested, “it is important to spend some time to reflect on what you have read, before moving on to the next segment of the program” (Participant 6). The same participant suggested that it was a good idea to log in once a week and complete tasks. Another participant stated that it was not sufficient to read only the content of the program, as self-effort and exercises were crucial for the program to be perceived as useful. It was also recommended that participants take notes as they read and completed segments of the program.

### 3.5 User satisfaction

#### 3.5.1 Opinions

Eight of the 10 participants expressed positive reactions to this type of treatment. Two participants who were initially sceptical became substantially motivated after using the program for the first time. One of them expressed, “I did not have much confidence in doing this myself” (Participant 6). However, three out of ten participants expressed more faith in face-to-face consultations.

One of the participants who was dissatisfied with the introduction to the program that was received was initially positive about trying the program, but lost interest upon feeling that the program's approach was not suitable for their own treatment needs. The participant felt that the therapist pushed the program upon them to avoid face-to-face consultations. This participant stated feeling deprived of much-needed in-person treatment.

Nine of the 10 participants were generally satisfied with the program. One participant expressed, “I think it is a very nice and straightforward program that contains a lot of useful information. So, I am very impressed” (Participant 2). This participant was originally unmotivated but became very engaged during the first experience, sitting for an hour and a half. Another participant expressed hope that this program would remain available and that as many people as possible would get the opportunity to use it. The same participant expressed, “I have had my anxiety disorder for many years without knowing what it actually is. If I only had known what I know now” (Participant 9).

#### 3.5.2 Perceived advantages and disadvantages

Commonly reported advantages among participants were low price, ability to engage peacefully, ability to read and answer questions, less effort required than travelling to a consultation, and ability to control the pace of engagement.

One dissatisfied participant thought the program was demanding, specifically with respect to the forms that had to be completed. This participant thought that the ‘*Assisted Self*-*Help*’ stress treatment program was more helpful than the anxiety program. Other reported disadvantages of the anxiety program were the loss of face-to-face consultations and a lack of therapist control and direction over participant engagement with the program.

### 3.6 Individual impacts

#### 3.6.1 Gain of knowledge

Eight of the 10 participants responded that the program contributed to increased insight into their anxiety disorder. Five participants commented that it was especially useful to receive explanations about the disorder's impact on their minds and bodies. One commented, “Whenever I had anxiety symptoms, I was very scared. But when I started reading, I started to understand a little bit. Yes, anxiety is normal, anxiety is not dangerous” (Participant 7). Another participant responded that the program contributed to better self-insight by instructing how to familiarize oneself with one's own mental health situation in a systematic way (i.e., use of forms). For example, the participant said, “Now I am more aware of self-securing strategies to avoid getting anxiety symptoms, such as getting enough sleep before going to work” (Participant 8).

#### 3.6.2 Competence to cope

All 10 participants expressed that the follow-up and guidance (or lack thereof) provided by their therapists affected their perceptions of the program's usefulness. Seven of the 10 participants thought that using the program had contributed to an increased ability to cope with their anxiety disorder. One commented, “I feel things have gone much better in the last few weeks after I started with it, absolutely” (Participant 2). All seven participants used the exposure therapy techniques they had learned through the program. Two of them felt that their ability to manage and cope with their symptoms had increased, although they were not 100% improved. One of the two noted less restlessness than before, while the other noted no longer having panic attacks. Both participants reported that the increase in knowledge afforded more control over and more confidence about their anxiety disorder. One of the participants commented that a change in mindset contributed to increased mastery. Nevertheless, another participant commented that it is important to continue working to maintain that control: “When I started with this … after 4–5 weeks, the anxiety disappeared for the first time in my life, and I thought now I'm fine. But then the panic attack came back again after 3 weeks, so it is important to keep it up” (Participant 9).

Among the three participants who did not think that the program contributed to increased mastery, one noted having prior knowledge and perhaps had not completed enough of the program to experience an effect. Another did not feel suited to the program, stating that exposure treatment was superior to reading. The remaining participants thought that if additional guidance (especially for exposure) had been received, it could have contributed to a better coping experience.

## 4 Discussion

The aim of this study was to conduct a user evaluation of a therapist-guided ICBT program (‘*Assisted Self*-*Help*’) for treating anxiety disorders using the updated D&M model for measuring IS success or effectiveness (Delone and McLean, 2003). Our study identified several important opinions from the participants regarding system quality, information quality, service quality, intention to use and use, user satisfaction, and individual impacts of the program. The results will be discussed in relation to the categories within the D&M model and their relationships.

### 4.1 System quality

Usability is a quality attribute that assesses the ease of use of a systems' user interface (Nielsen, 2012). Utility, or a systems provision of needed functionalities and characteristics, is considered an important aspect of usability (Nielsen, 2012). In this study, few usability issues were identified, but such issues align with important quality attributes for user interface design (i.e., usability heuristics), such as “help and documentation,” “flexibility and efficiency,” and “visibility of system status” (Nielsen, 1994). For example, in the studies by Vlaescu et al. (2016) and Johansson et al. (2015), some participants were displeased by the automated measurements of participant progress within each module—an issue of flexibility and efficiency. Despite the above issues, overall, the participants seemed quite satisfied with the system quality. However, these results are not quite in line with the results of a usability evaluation conducted by Yogarajah et al. (2020). In that study, several usability issues were identified with ‘*Assisted Self*-*Help*’ and four other Scandinavian guided ICBT programs. The lack of agreement between that study and the present study might be due to the fact that their evaluation was a heuristic evaluation conducted by one researcher specializing in information communication technology. Further, the success criteria of their evaluation were based on universal design principles, such as the web context accessibility guidelines (WCAG 2.0). The intention of such guidelines is to define how to make web content more accessible to people with visual, auditory, physical, speech, cognitive, language, learning, and neurological disabilities. The lack of agreement of study results indicates that it may be useful to conduct a usability evaluation of the ‘*Assisted Self*-*Help*’ program and its different platforms with end users in a usability laboratory. Design, layout, structure, usability, and interactivity have been considered by ICBT users as essential elements for maintaining engagement throughout the course (Health Quality Ontario, 2019). Thus, developers of digital solutions, including guided ICBT, should involve users in the development process to ensure that the solutions meet the needs and preferences of individual user groups (Andersson, 2018).

According to Petter et al. (2008), system quality may have moderate associations with individual impacts. This was partly supported by our participants. Even if our results indicated a strong relationship between system quality and satisfaction, in line with Petter et al. (2008), three participants did not perceive that the program had been useful. These opinions correspond with evidence showing no empirical evidence of the association of system quality with individual impact (Shim and Jo, 2020).

### 4.2 Information quality

Certain disabilities, as well as an inability to understand complex written information or language, may present barriers to using guided ICBT (Health Quality Ontario, 2019; Johansson et al., 2015). None of the participants mentioned having disabilities, but one participant of foreign origin had trouble understanding some of the content. This required more thorough follow-up and guidance from their therapist. Otherwise, all participants seemed satisfied with the information quality. Even the most experienced and knowledgeable participants thought the theoretical content on anxiety disorder physiology and real-life patient stories were particularly relevant and useful. This aligns with evidence showing that ICBT is beneficial for patients because it provides easy access to educational material (Health Quality Ontario, 2019). Further, the participants perceived it particularly useful to personally identify with the content in the program. Thus, the results from this study are in line with Delone and McLean (2003) and Petter et al.'s (2008) suggestions that information quality has a strong association with individual impacts. This is also supported in a study using the D&M model to evaluate the benefits of online health information sites (Shim and Jo, 2020).

In contrast to ‘*Assisted Self*-*Help*’, other European guided ICBT programs have had difficulty tailoring programs to individual patients (Folker et al., 2018). Participants appreciated that ‘*Assisted Self*-*Help*’ enables users to choose between different anxiety disorders, which further tailors the content of the program. However, some participants expressed that it could be useful to receive more guidance on which program was most suitable for their condition. This result may indicate that service quality can indirectly influence information quality through the content provided in the program. However, such relationships do not seem to have been examined (Jeyaraj, 2020).

### 4.3 Service quality

Known barriers to using guided ICBT include negative attitudes or scepticism towards internet-based treatment and lack of awareness of this type of treatment option (Folker et al., 2018). Similar to other studies (Johansson et al., 2015), participants reported being under-informed about the concept of guided ICBT and ‘*Assisted Self*-*Help*’ specifically. As suggested by one of the participants, external communication and marketing of guided ICBT services is an important vehicle for the recruitment of patients (Folker et al., 2018; Hadjistavropoulos et al., 2017). This is supported by the fact that self-referral to guided ICBT has been found to be the most viable and promising referral model, as patients who actively chose guided ICBT reported more motivation than referred patients. It has also been proposed that self-referral may ease access and reduce waiting times in the system (Folker et al., 2018). Guided ICBT services should be promoted through different dissemination channels, including the municipality's website (as suggested by one of the participants), social media, leaflets, and educational videos.

Furthermore, participants reported difficulty being convinced about the benefits of guided ICBT if the therapist was not sufficiently knowledgeable about ICBT. One important reason why patients fail to adhere to guided ICBT is that they have been provided with insufficient information before starting the program and have thus been unaware of the full scope of this type of treatment (Johansson et al., 2015). Our results indicate that therapists should be provided with sufficient information about the guided ICBT program they offer to their patients. Additionally, user manuals and instruction videos should be offered to both therapists and patients. This may ensure both the quantity and quality of the information provided.

Therapeutic alliance is an important element in guided ICBT and seems to be associated with treatment outcomes (Pihlaja et al., 2018; Shim et al., 2017; Stjerneklar et al., 2019). Results from our study also support this point. Participants in this study reported that the guidance and support (or lack thereof) received from their therapists influenced their motivation and self-effort and their perceived usefulness of guided ICBT. The importance of guidance and support alongside is also elaborated in several qualitative studies about users' experiences with ICBT programs (Pedersen et al., 2020; Walsh and Richards, 2017; Knowles et al., 2014).

Positive treatment outcomes have been observed more often when follow-up and guidance have been provided at regular fixed intervals rather than on an as-needed basis (Shim et al., 2017). This observation was partly supported by the participants in the present study. Four out of the 10 participants had weekly appointments, while two participants had biweekly appointments. Nevertheless, the results of this study also support the notion that patients have unique guidance and support needs (Andersson, 2018). One participant who made infrequent appointments expressed that the program was useful, even without regular guidance, as long as the participant knew that the therapist was available if guidance and support were ever needed.

The influence of service quality on system use or service quality on user satisfaction has not been frequently examined or found to be significant in previous studies (Jeyaraj, 2020). However, in accordance with suggestions from the updated D&M model (Delone and McLean, 2003), our study identified such influence. As proposed by Jeyaraj (2020), we think this could be explained by the context in which the model has been applied and the service provision.

### 4.4 Intention to use and use

Intention to use (attitude) and its links with actual system use (behaviour) have been difficult to measure (Delone and McLean, 2003; Jeyaraj, 2020). Consequently, many researchers choose to only apply system use in the application of the D&M model. However, the results from this study indicate that service quality may strongly influence motivation, effort, and perceived usefulness. Further, our results also support the proposition that experience of positive or negative benefits will lead to increased or decreased intention to use and user satisfaction (Delone and McLean, 2003).

Evidence has shown a positive correlation between time spent on internet-delivered treatment programs and clinical effects (Farrer et al., 2014). The time spent on the ‘*Assisted Self*-*Help*’ program and the completion time varied among the participants. In contrast to findings by Farrer et al. (2014), one of the participants in the present study claimed that it was not the time spent that was important, but rather the reflections on the content between sessions. This is supported by Johansson et al. (2015), who suggested that participants may need time to process the knowledge gained between modules. As such, a treatment plan that is too fixed may cause nonadherence among guided ICBT users. This assertion is supported by one of the present study's least satisfied participants, who expressed not having sufficient time to finish the modules before therapy appointments. The above results are supported by Delone and Mclean (2003), who proposed that system use has to be appropriate to achieve expected impacts.

As regards module completion, one participant had completed only about half of the program and was thus unsure about the ultimate usefulness of the program at the time of interview. This is in line with evidence showing that the number of completed modules is a predictor of treatment response (Stjerneklar et al., 2019). Most of the participants had completed the program, but the COVID-19 pandemic extended the completion period for several participants. Nevertheless, those participants had initially planned to finish the program. Several of the participants who completed the program also intended to repeat the program or complete other programs in the ‘*Assisted Self*-*Help*’ resource. This may be related to their satisfaction with the program but can also be explained by the fact that the ‘*Assisted Self*-*Help*’ program only lasts about 6 weeks. For comparison, similar resources in Europe last between 10 and 20 weeks (Folker et al., 2018). Evidence has shown that the duration of guided ICBT programs and individual time constraints are among the common reasons for non-completion of guided ICBT programs (Johansson et al., 2015). Similarly, the duration of a program or the time needed to complete a digital program (i.e., efficiency) is viewed as a usability attribute that may influence a person's motivation and intention to use the technology (Nielsen, 2012).

### 4.5 User satisfaction

‘*Assisted Self*-*Help*’ offers synchronous communication options, such as face-to-face, phone-based, or video-based appointments. However, due to the COVID-19 pandemic, the participants were offered only phone-based or video-based appointments. At the time of data collection, most participants had phone-based appointments, and a few were video-based. However, similar to participants in other studies (Johansson et al., 2015; Health Quality Ontario, 2019), several of the participants in this study expressed a preference for face-to-face appointments.

In line with other studies (Soucy and Hadjistavropoulos, 2017), the participants in this study reported advantages with guided ICBT, including flexibility and convenience. This was based on guided ICBT requiring less effort and affording participants the ability to avoid social interaction. Further, similar to the Health Quality Ontario study (Health Quality Ontario, 2019), participants perceived it advantageous to have access to learning materials at all times and to have control over the pace and length of sessions.

The results also showed that instructions and guidance (or lack thereof) from the therapist were important factors impacting adherence and satisfaction. This is in line with prior research (Soucy and Hadjistavropoulos, 2017). However, dissatisfaction and non-adherence to guided ICBT may also be caused by treatment factors (e.g., workload and program complexity) or personal factors (e.g., perceived mismatch between treatment process and personal prerequisites regarding daily routines or perceived language skills and treatment expectations) (Johansson et al., 2015). Two of the participants who were not satisfied with the guidance and support they received mentioned workload and personal prerequisites as stressors. Not being suited to guided ICBT is a commonly reported problem among patients who self-refer to or are referred by GPs (Folker et al., 2018). This mismatch may also be explained by the fact that the ‘*Assisted Self*-*Help*’ program is intended only to treat mild-to-moderate anxiety disorders rather than severe anxiety or combinations of other diagnoses.

In line with results from the critical metareview of Jeyaraj (2020), all three quality dimensions (system quality, information quality, and service quality) impacted our users satisfaction. Application of the D&M model in the evaluation of an e-learning system found similar results as in our study—that individual characteristics may influence the different dimensions and individual impacts (Cidral et al., 2018).

### 4.6 Net benefits

The most common relationships that have been examined in the application of the D&M model are between system quality and information quality and net benefits (Jeyaraj, 2020). However, a cornerstone of health promotion is empowering individuals to ensure their own mental health and well-being (World Health Organisation, 2019; United Nations, 2020). This is in line with the updated D&M model (Delone and McLean, 2003) and other studies (Pihlaja et al., 2018; Shim et al., 2017; Stjerneklar et al., 2019), suggesting that support from different service providers may influence individual impact. As indicated by the results of this study, an educational approach (i.e., learning exposure techniques and the physiology of anxiety disorders) alone is not sufficient for the improvement of an anxiety disorder. Active participation from all involved in the process, including the therapist (Bunton and Macdonald, 2002), is necessary. This is supported by the notion that several factors may influence treatment uptake and user adherence to guided ICBT (Soucy and Hadjistavropoulos, 2017). Thus, the results are in line with the D&M success model (Petter et al., 2008), as the model proposes that the six dimensions of success are interrelated rather than independent, with each dimension necessary to attain ICBT success.

### 4.7 Strengths and limitations of the study

Despite growing evidence supporting guided ICBT's effects and individual predicting factors of treatment response (Andersson, 2018; Stjerneklar et al., 2019), more research is needed to investigate and describe how guided ICBT programs can be implemented successfully (Drozd et al., 2016; Folker et al., 2018). Thus, one of the strengths of this study is that it introduced a success model with several dimensions necessary for achieving positive effects from information systems like guided ICBT. In addition, this study demonstrated how this success model can be employed to explore how different D&M dimensions influence patients' perceived usefulness of a specific guided ICBT program. To increase the validity and reliability of the results, the main categories of the directed analysis were based on the dimensions of the D&M model. A limitation of using a directed approach is that it can blind researchers to contextual aspects and bias identification of other possible main categories or subcategories in the text (Hsieh and Shannon, 2005). However, the authors kept an open mind during the analysis process in case other main themes or subthemes occurred from the data.

The present study has certain limitations. For example, the sample size was limited. Fortunately, the data collected from the 10 participants reached a point where no new information was discovered—a state called data saturation (Polit and Beck, 2010). As several participants finished the program a few months before the interview, their evaluations of the program relied on their memories. Nevertheless, most of the participants' maintained access to the program and were able to log in and refresh the information if they failed to remember.

In this study, the therapist, rather than the organisation, was viewed as the service provider. An optimal view would have involved gathering more information about how guided ICBT was implemented in the particular departments of the organisation where the therapists work and what resources, information, and training the therapists received on how to use guided ICBT. For example, Folker et al. (2018) showed that therapists experience implementation issues stemming from a lack of information, user manuals, and training related to different guided ICBT programs. Gathering such information may help explain the degree of follow-up, guidance, and support that different patients received in this study. These factors were not explored, as this study employed a patient view rather than an organisational view.

Trustworthiness values in the qualitative analyses, such as credibility, dependability, and transferability (Graneheim and Lundman, 2004), were protected by the chosen procedure for qualitative design. Criteria for credibility, which are understood as keeping the focus of the project, were met by choosing participants who were relevant to the aim of this study. Dependability was met using the same interview guide for all participants, and no major changes were made during the data collection. Moreover, the main author conducted all the interviews. The main author has expertise in health informatics research and is familiar with using different models to measure IS success and user acceptance of technology. The second author has expertise in literature searches and qualitative analysis. Both authors are nurses. However, one limitation may be that none of the authors specialize in mental health or anxiety disorders. By thoroughly describing the context, the participants, and research process transferability, it should be possible to achieve the same standard as for similar studies. Thus, we argue that the qualitative part of the study meets the criteria for trustworthiness.

## 5 Conclusion

In line with the D&M model, the results in this study indicate that a therapist-guided ICBT program's user-friendliness and high-quality educational content are not alone sufficient for a guided ICBT program to be perceived as useful. It is necessary that proper instruction, follow-up, guidance, and support from therapists be in line with patients' individual needs. Further, a strong working alliance must be created during the first appointment and maintained throughout the treatment process. These results provide useful information for a forthcoming randomized controlled study that will evaluate the effect of the therapist-guided ICBT program ‘*Assisted Self*-*Help*’.

A cornerstone of health promotion is empowering individuals to ensure their own mental health and well-being. This requires active participation from all involved in the process, including the therapist. We propose that guidelines should be established for developing and implementing therapist-guided ICBT programs to ensure that important dimensions are considered, which is necessary for patients to perceive this type of treatment as useful.

## Declaration of competing interest

This research project was commissioned by Assistert Selvhjelp AS and conducted by the authors on behalf of the Centre for e-Health at the University of Agder. Assistert Selvhjelp AS had no role in the study design, collection, analysis or interpretation of data, writing the manuscript, or publication of this manuscript. There has been no financial relationship with Assistert Selvhjelp AS. The project was funded by the main author's own departmental funds at the Department of Health and Nursing Science, University of Agder.
